# Identification of liver *CYP51* as a gene responsive to circulating cholesterol in a hamster model

**DOI:** 10.1017/jns.2016.3

**Published:** 2016-03-30

**Authors:** Haiqiu Huang, Zhuohong Xie, Wallace Yokoyama, Liangli Yu, Thomas T. Y. Wang

**Affiliations:** 1 Diet, Genomics and Immunology Laboratory, USDA-ARS, Beltsville, MD 20705, USA; 2 International Chemistry Testing, Milford, MA 01757, USA; 3 Processed Foods Research, USDA-ARS, Albany, CA 94710, USA; 4 Department of Nutrition and Food Science, University of Maryland, College Park, MD 20742, USA

**Keywords:** Circulating cholesterol, *CYP51*, Hamsters, Hypercholesterolaemia, ABCG5/, ATP-binding cassette subfamily G member 5/8, CA, cholestyramine, CYP51, lanosterol 14α-demethylase, CYP7A1, cholesterol 7α-hydroxylase, HF, high-fat, HF + CA, high-fat + cholestyramine, HMG, 3-hydroxy-3-methyl-glutaryl, IPA, Ingenuity Pathway Analysis, LXRα, liver X receptor α, RXR, retinoid X receptor, SREBP, sterol regulatory element-binding protein

## Abstract

Hypercholesterolaemia is a risk factor for CVD, which is a leading cause of death in industrialised societies. The biosynthetic pathways for cholesterol metabolism are well understood; however, the regulation of circulating cholesterol by diet is still not fully elucidated. The present study aimed to gain more comprehensive understanding of the relationship between circulating cholesterol levels and molecular effects in target tissues using the hamster model. Male golden Syrian hamsters were fed with chow or diets containing 36 % energy from fat with or without 1 % cholesteyramine (CA) as a modulator of circulating cholesterol levels for 35 d. It was revealed that the expression of lanosterol 14α-demethylase (*CYP51*) instead of 3-hydroxy-3-methyl-glutaryl (HMG)-CoA reductase mRNA expression was responsive to circulating cholesterol in hamsters fed hypercholesterolaemic diets. The high-fat diet increased circulating cholesterol and down-regulated *CYP51*, but not HMG-CoA reductase. The CA diet decreased cholesterol and increased *CYP51* expression, but HMG-CoA reductase expression was not affected. The high-fat diet and CA diet altered the expression level of cholesterol, bile acids and lipid metabolism-associated genes (LDL receptor, cholesterol 7α-hydroxylase (*CYP7A1*), liver X receptor (*LXR*) α, and ATP-binding cassette subfamily G member 5/8 (*ABCG5/8*)) in the liver, which were significantly correlated with circulating cholesterol levels. Correlation analysis also showed that circulating cholesterol levels were regulated by LXR/retinoid X receptor and PPAR pathways in the liver. Using the hamster model, the present study provided additional molecular insights into the influence of circulating cholesterol on hepatic cholesterol metabolism pathways during hypercholesterolaemia.

Hypercholesterolaemia is one of the major factors contributing to the onset and progression of CVD, the leading cause of death in industrialised societies^(^^1^^)^. Significant progress has been made in understanding of cholesterol metabolism since Goldstein & Brown's seminal work^(^^2^^)^. Experimental and epidemiological studies have indicated that increased dietary intake of fat and cholesterol is a major contributor to hypercholesterolaemia in the Western world^(^^3^^,^^4^^)^. Dysregulation of cholesterol *de novo* synthesis or cholesterol deposition can also result in elevation in circulating cholesterol level^(^^5^^,^^6^^)^. 3-Hydroxy-3-methyl-glutaryl (HMG)-CoA reductase, the rate-limiting enzyme in the cholesterol synthesis pathway, and LDL receptor have been the main candidates of cholesterol metabolism in previous research^(^^7^^–^^10^^)^. Current understanding of cholesterol biosynthesis and deposition indicates three main pathways: (1) regulation of HMG-CoA reductase activity and levels; (2) regulation of excess intracellular non-esterified cholesterol through the activity of acyl-CoA:cholesterol acyltransferase; and (3) regulation of plasma cholesterol levels via LDL receptor-mediated uptake and HDL-mediated reverse transport^(^^7^^)^. In addition, pathways involved in bile acid metabolism and cholesterol absorption can also influence the circulating levels of cholesterol as well as its deposition^(^^11^^,^^12^^)^. However, the majority of previous studies have been concentrated on regulation and reduction of disease-state cholesterol levels; the molecular events related to changes in circulating cholesterol level are less understood.

The hamster is widely accepted as a suitable animal model for studying human cholesterol metabolism. The lipid profiles and susceptibility to dietary cholesterol of the golden Syrian hamster (*Mesocricetus auratus*) are similar to those of man^(^^13^^–^^15^^)^. In man and the hamster, LDL is the dominant lipoprotein, whereas HDL is the major plasma lipoprotein in other animal models (mouse, rat, dog and monkey)^(^^16^^,^^17^^)^. The hamster also exhibits similar cholesteryl ester transfer protein activity as man, which is absent in the rat^(^^16^^–^^18^^)^.

The present study aimed to gain more comprehensive understanding of relationship between circulating cholesterol levels and molecular effect in target tissues using the hamster model. A high-fat (HF) diet or the cholesterol-lowering drug cholestyramine (CA), which was given at 1 %, which is comparable with common prescription^(^^19^^)^, was used to modulate plasma cholesterol in the presnt study. Biochemical and molecular changes in cholesterol and lipid metabolism were determined to elucidate the relationship between plasma, liver and faecal cholesterol and lipid levels, as well as the regulation of the expression level of cholesterol- and lipid metabolism-related genes in the liver.

## Materials and methods

### Animals and diets

Male golden Syrian hamsters (approximately 80 g, LVG strain; Charles River) were given free access to water and rodent chow to acclimatise to the environment for 1 week prior to the experiment. For the experiment, hamsters were randomised into three groups of ten and fed one of the following: a HF diet (36 % energy from fat diet with 1·4 g cholesterol/kg diet), a HF diet containing CA (HF + CA) (36 % energy from fat diet with 1·4 g cholesterol/kg diet containing 1 % CA), or chow (with 0·05 g cholesterol/kg diet, 8728C Teklad Certified Rodent Diet; Harlan Laboratories, Inc.). Diets consisted of 18 % protein, 45 % carbohydrate, and 36 % fat on an energy basis. The hamster and human HED (human equivalent dose) has a *k*_m_ factor of 5:37^(^^20^^)^, and the 1 % CA used in hamsters (about 120 g) is calculated based on 5 g for a 60 kg adult human. Diet compositions are listed in Table 1. The experimental diets were formulated and purchased from Research Diets. Animals (ten per group) were fed with the respective diet for 5 weeks with water available *ad libitum*. Food intake and body weight were recorded twice per week. The animal use and care protocol (protocol no. 10–014) for this study was reviewed and approved by the US Department of Agriculture (USDA), Agricultural Research Service, Beltsville Area Animal Care and Use Committee (BAACUC).

**Table 1.** Diet content

	High fat*		High fat + cholestyramine*		Chow†	
	g %	Energy %	g %	Energy %	g %	Energy %
Protein	21·0	18·0	21·0	18·0	24·3	32·0
Carbohydrate	52·0	45·0	52·0	45·0	40·2	54·0
Fat	19·0	36·0	19·0	36·0	4·7	14·0
Others					23·8	
Total		100·0		100·0		100·0
Energy						
kcal/g kJ/g	4·58 19·16		4·58 19·16		3·00 12·55	
Ingredient	g	kcal	g	kcal	g	kcal
Casein	222·0	888·0	222·0	888·0		
dl-Methionine	3·0	12·0	3·0	12·0		
Maize starch	453·0	1812·0	453·0	1812·0		
Maltodextrin	100·0	400·0	100·0	400·0		
Sucrose	0·0	0·0	0·0	0·0		
Cellulose	53·0	0·0	53·0	0·0		
Maize oil	100·0	900·0	100·0	900·0		
Butter	80·0	720·0	80·0	720·0		
Menhaden oil	20·0	180·0	20·0	180·0		
Mineral mix	45·0	40·0	45·0	40·0		
Choline bitartrate	3·0	0·0	3·0	0·0		
Cholesterol	1·5	0·0	1·5	0·0	0·05	0·0
Cholestyramine	0·0	0·0	10·9	0·0	0·0	0·0
Total	1080·5	4952·0	1091·4	4952·0	1000·0	3000·0

* The high fat and high fat + cholestyramine diets were formulated and purchased from Research Diets.

† The chow diet was purchased from Harlan Laboratories.

### Plasma and tissue collection

Hamsters were subjected to 12 h fasting prior to killing and anaesthetised with CO_2_. Blood was collected by cardiac puncture with syringes previously rinsed with potassium EDTA solution (15 %, w/v), and plasma was separated after centrifugation at 1300 ***g*** for 15 min at 4°C. Livers and flank adipose tissues were collected, weighed, and one part of the tissue was immediately frozen in liquid N_2_ for analysis; the other part was preserved in RNALater RNA stabilisation solution (Ambion) and kept at −80°C.

### Plasma lipoprotein analysis

Plasma lipoprotein cholesterol concentration was determined by size exclusion chromatography as previously described^(^^21^^)^. Briefly, an Agilent 1100 chromatograph was employed with a post-column derivatisation reactor, consisting of a mixing coil (1615–50 Bodman) in a temperature-controlled water jacket (Aura Industrials). A Hewlett-Packard (Agilent) HPLC pump 79851-A was used to deliver cholesterol reagent (Roche Diagnostics) at a flow rate of 0·2 ml/min. Bovine cholesterol lipoprotein standards (Sigma Aldrich) were used to calibrate the signal on the basis of peak areas. A quantity of 15 µl of plasma was injected via an Agilent 1100 auto sampler onto a Superose 6HR HPLC column (Pharmacia LKB Biotechnology). The lipoproteins were eluted with a pH 7·0 buffer solution containing 0·15 m-sodium chloride and 0·02 % sodium azide at a flow rate of 0·5 ml/min. Plasma lipoprotein concentration was calculated based on a standard curve.

### Hepatic lipid extraction

Livers were excised and immediately frozen in liquid N_2_, and then stored at −80°C prior to analysis. The extraction was a modification of the Folch method^(^^22^^,^^23^^)^. Approximately 0·15 g frozen liver were minced and transferred into a test tube. Then 6 ml of chloroform–methanol (2:1, v/v) were added, followed by a 2 min homogenisation and 30 s of sonication at 30 % power level. Samples were then incubated with shaking for 2 h on a platform shaker. After incubation, 2 ml of double distilled water were added. Samples were then centrifuged at 500 ***g*** for 20 min. After centrifugation, the bottom layer was carefully aspirated into a new test tube and incubated overnight. It was then filtered through a 0·22 µm filter and dried by stream of N_2_. The dried lipid was weighed and re-dissolved in isopropanol with 10 % Triton X-100 and used for TAG and cholesterol analysis as described below.

### TAG, total cholesterol and non-esterified cholesterol in liver

Hepatic TAG, total cholesterol and non-esterified cholesterol were enzymically determined using commercial kits (Triglyceride-SL, Genzyme Diagnostics PEI Inc.; Cholesterol E and Free Cholesterol E, Wako Chemicals) following manufacturers’ protocols.

### Faecal bile acids and cholesterol extraction

Faecal bile acids and cholesterol were extracted using a modified protocol^(^^24^^)^. Faecal samples were collected during a 48 h period on days 33–35 after the initiation of the experiment. The samples were lyophilised, pulverised using a pestle and mortar, and weighed. A dried faecal sample (0·10 g) was hydrolysed in 1·0 ml of 2 m-KOH at 50°C for 5 h. The cooled mixture was then extracted with two 6 ml portions of diethyl ether to remove non-saponifiable components. Subsequently, 1 ml of 20 % sodium chloride followed by 0·2 ml of 12 m-hydrochloric acid were added to the remaining mixture. The acidified mixture was extracted with two 6 ml portions of diethyl ether and the pooled ether extracts were evaporated by N_2_ and redissolved in 0·5 ml of ethanol. The samples were used for faecal bile acid and cholesterol determination as follows.

### Faecal bile acids and cholesterol analysis

Faecal bile acid content was determined using a kit from Sigma-Aldrich as previously described^(^^25^^)^. β-Nicotinamide adenine dinucleotide hydrate (NAD), nitroblue tetrazolium chloride (NBT), diaphorase, 3α-hydroxysterol dehydrogenase (3α-HSD) and cholic acid were obtained from Sigma-Aldrich. NAD, NBT, diaphorase and 3α-HSD were prepared in 0·01 m-phosphate buffer at pH 7·0. The reaction mixture included 40 µl of sample or standard with 4 µl of Triton X-100, 50 µl of NAD (2·5 mm), 50 µl of NBT (0·61 mm), 50 µl of diaphorase (625 U/l) and 50 µl of 3α-HSD (625 U/l). The mixture was incubated for 60 min at ambient temperature, after which 40 µl of phosphoric acid (1·33 m) were added to stop the reaction. The absorbance of each reaction mixture was measured at 530 nm. Cholic acid in ethanol was used to generate a standard curve and the amount of faecal bile acid obtained was determined using the standard curve. Cholesterol was determined by the same assay as for liver described above.

### Total RNA isolation, cDNA synthesis and gene expression analysis

To determine the gene expression changes, liver preserved in RNALater was cut into 0·1 to 0·2 g pieces and homogenised using a Precellys 24 (Bertin Technologies). The RNeasy Mini Kit (Qiagen) was used for total RNA isolation from liver. The AffinityScript cDNASynthesis kit from Agilent was used to reverse transcribe complementary DNA. Real-time PCR was performed on an Applied Biosystems 7900HT Sequence Detection System using Fast SYBR Green Master Mix (Life Technologies). Primers used in this study are listed in Table 2. Relative mRNA expression levels were calculated using the ΔCt method^(^^26^^)^. Glyceraldehyde-3-phosphate dehydrogenase (GAPDH) expression was used as the housekeeping gene for calculations.

**Table 2.** Sequences of real-time PCR primers (SYBR Green primers)

Genes	Direction	Sequence (5′–3′)
*GAPDH*	Forward	GAACATCATCCCTGCATCCA
	Reverse	CCAGTGAGCTTCCCGTTCA
*HMGCR*	Forward	CGAAGGGTTTGCAGTGATAAAGGA
	Reverse	GCCATAGTCACATGAAGCTTCTGTA
*LDLR*	Forward	TGAGGAACATCAACAGCATAAAC
	Reverse	ATCCTCCAGGCTGACCATCTGT
*LXRα*	Forward	ATTGCCATCAGCATCTTCTCT
	Reverse	GCATCCGTGGGAACATCAGT
*PPARα*	Forward	CTCCACCTGCAGAGCAACCA
	Reverse	CGTCAGACTCGGTCTTCTTGAT
*ABCG5*	Forward	TGATTGGCAGCTATAATTTTGGG
	Reverse	GTTGGGCTGCGATGGAAA
*ABCG8*	Forward	TGCTGGCCATCATAGGGAG
	Reverse	TCCTGATTTCATCTTGCCACC
*CYP7A1*	Forward	GGTAGTGTGCTGTTGTATATGGGTTA
	Reverse	ACAGCCCAGGTATGGAATCAAC
*CYP51*	Forward	GAGAGAAGTTTGCCTATGTGCC
	Reverse	TGTAACGGATTACTGGGTTTTCT
*SREBP*	Forward	GCGGACGCAGTCTGGG
	Reverse	ATGAGCTGGAGCATGTCTTCAAA
*FAS*	Forward	AGCCCCTCAAGTGCACAGTG
	Reverse	TGCCAATGTGTTTTCCCTGA
*ACOX*	Forward	TTACATGCCTTTGTTGTCCCTATC
	Reverse	CGGTAATTGTCCATCTTCAGGTA

*GAPDH*, glyceraldehyde 3-phosphate dehydrogenase; *HMGCR*, 3-hydroxy-3-methyl-glutaryl-CoA reductase; *LDLR*, LDL receptor; *LXR*, liver X receptor; *ABCG5/8*, ATP-binding cassette subfamily G member 5/8; *CYP7A1*, cholesterol 7α-hydroxylase; *CYP51*, lanosterol 14α-demethylase; *SREBP*, sterol regulatory element-binding protein; *FAS*, fatty acid synthase; *ACOX*, acyl-CoA oxidase.

### Pathway analysis and prediction

Expression of cholesterol and bile acid metabolism genes in the HF and HF + CA groups was subjected to core pathway analysis using the Ingenuity Pathway Analysis (IPA) software (Ingenuity Systems). Using IPA and its accompanying interaction database, a list of canonical pathways was determined based on core analysis of significance of changes in these genes. IPA also generated a network of the cholesterol and bile acid metabolism genes and upstream regulator genes, in which their potential interactions were depicted.

### Statistical analysis

All end point assays for each sample were conducted in triplicate and the average was used for group analysis, data for each treatment group are presented as means with their standard errors. Significance level of differences in means was detected using one-way ANOVA and Tukey's test. Correlation between plasma lipoproteins, hepatic lipid, cholesterol, and bile acids, faecal cholesterol and bile acids, and hepatic gene expression levels were calculated using the two-tailed Pearson correlation method. Linear regression analysis of ATP-binding cassette subfamily G member 5 (ABCG5), ATP-binding cassette subfamily G member 8 (ABCG8), cholesterol 7α-hydroxylase (CYP7A1), hepatic lipid content, and faecal bile acids, and polynomial regression analysis of lanosterol 14α-demethylase (CYP51), and total lipoprotein and hepatic cholesterol, were performed using Graphpad Prism 6 (2012; Graphpad Software). Statistics were analysed using IBM SPSS Statistics 19.0 (2010; IBM Corporation). Statistical significance was defined at *P* ≤ 0·05.

## Results

### Food intake and body weight

**Fig. 1.**
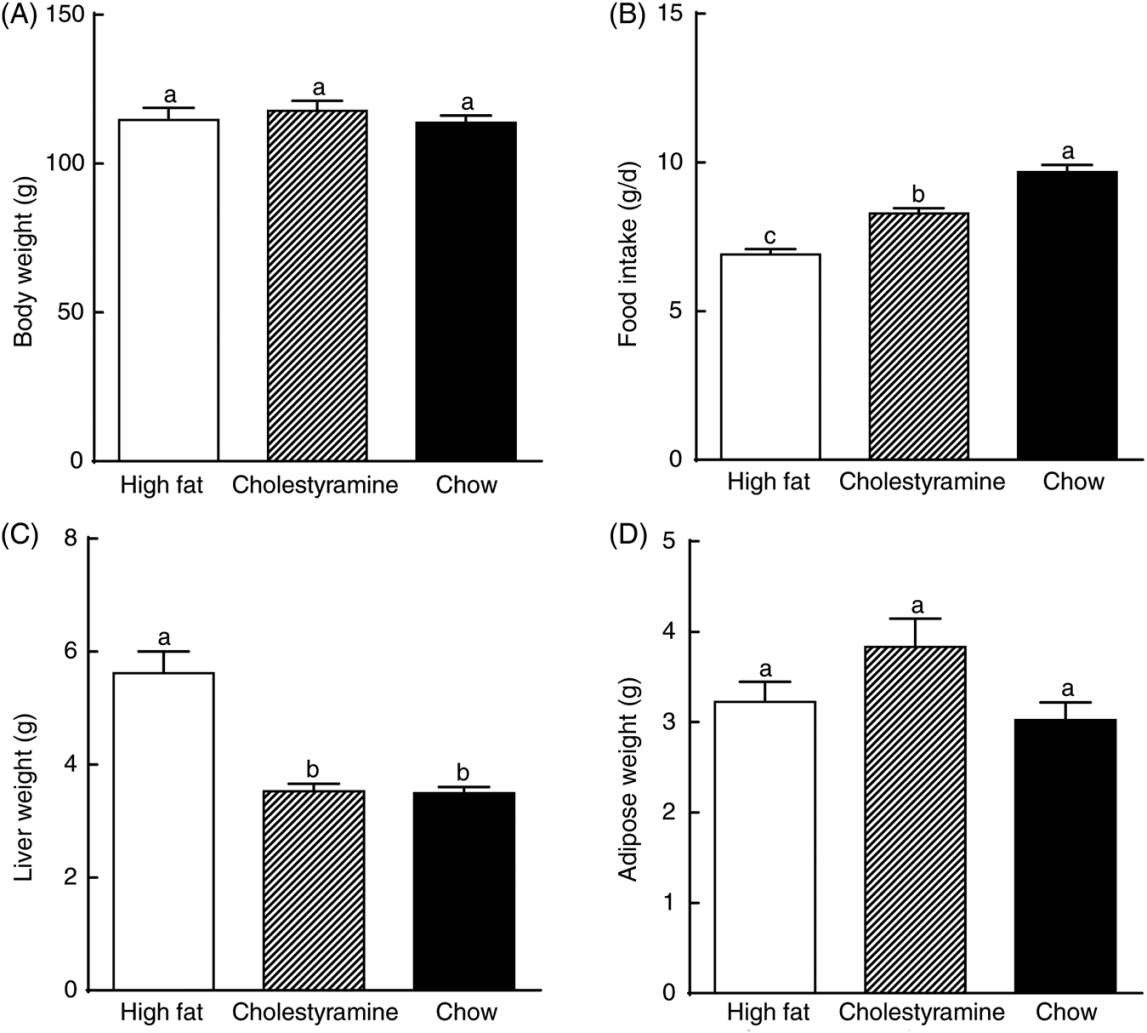
Food intake, and body and tissue weights. Hamster body (A), liver (C) and adipose (D) weights after 5-week feeding period. Food intake (B) was calculated as average daily intake. Values are means (*n* 10), with standard errors represented by vertical bars. ^a,b,c^ Mean values with unlike letters were significantly different (*P* ≤ 0·05).

No differences in body weight were observed among the diet groups throughout the 5-week feeding period (Fig. 1(A)). Food intake was significantly different among the three diet groups. The chow diet group had the highest food intake and the HF group consumed the least (Fig. 1(B)). However, due to the higher fat content, HF groups recorded higher energy intake. The HF + CA group had the highest energy intake of 37·97 (se 2·55) kcal/d (158·87 (se 10·67) kJ/d), and energy intake for the HF group was 31·68 (se 2·43) kcal/d (132·55 (se 10·17 kJ/d), while 29·07 (se 2·17 kcal/d (121·63 (se 9·08) kJ/d) for the chow group was the least despite the higher food intake. The HF group had the highest liver weight, and the HF + CA group had a liver weight similar to that of the chow diet group (Fig. 1(C)). There were no differences in weight of adipose tissue across the diet groups (Fig. 1(D)).

### Plasma lipoprotein cholesterol content

**Fig. 2.**
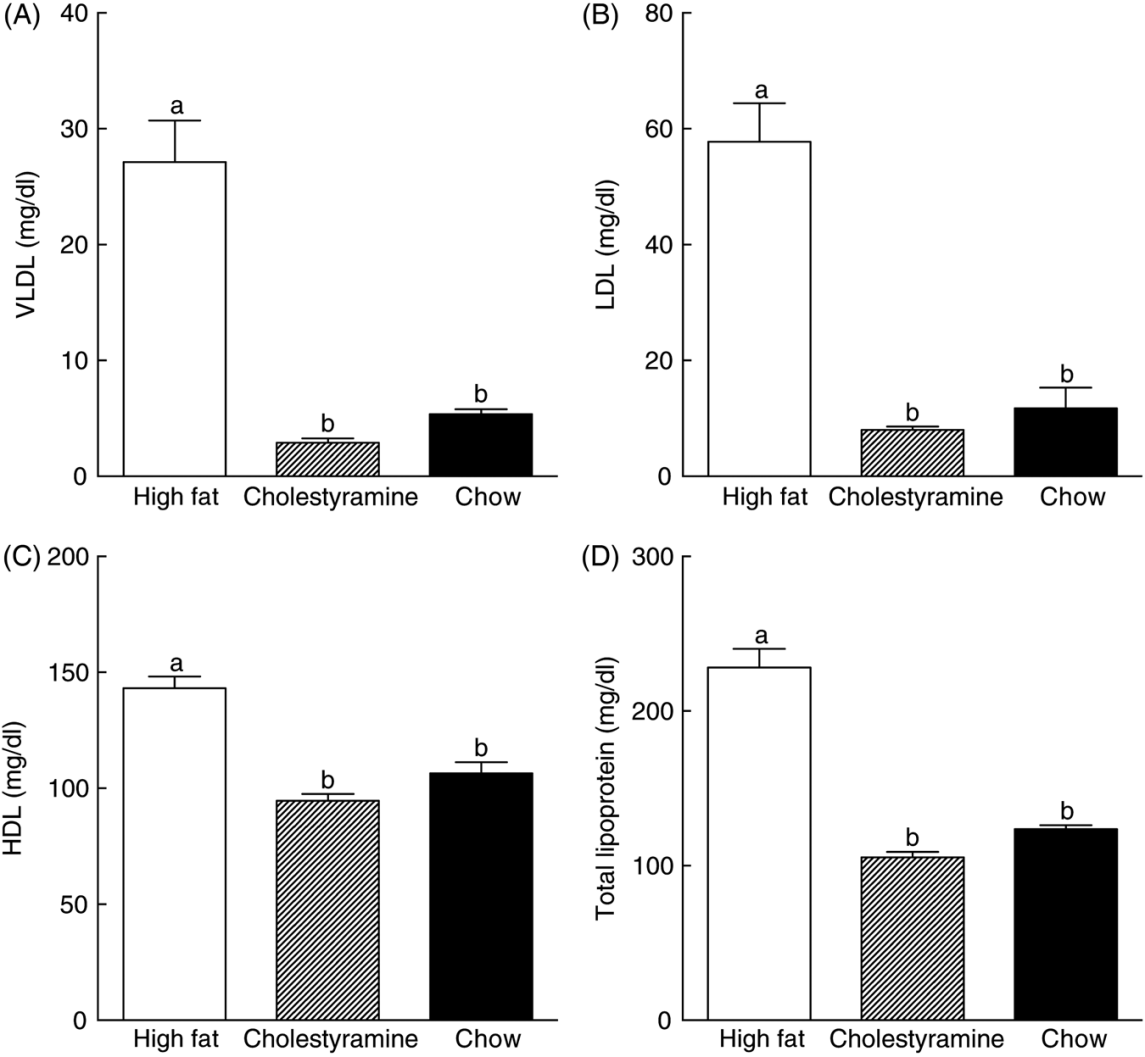
Effects of cholestyramine supplementation on plasma lipoprotein cholesterol levels. Hamster plasma from different diet groups was harvested and plasma lipoprotein cholesterol concentrations were determined by size exclusion chromatography as described in Materials and methods. (A) VLDL, (B) LDL, (C) HDL and (D) total lipoprotein. Values are means (*n* 10), with standard errors represented by vertical bars. ^a,b^ Mean values with unlike letters were significantly different (*P* ≤ 0·05). To convert mg/dl to mmol/l, multiply by 0·0259.

Consumption of the HF diet significantly elevated VLDL (407 %), LDL (394 %), HDL (35 %) and total lipoprotein levels (85 %) in hamster plasma compared with the chow diet group (Fig. 2). Animals on the HF + CA diet showed significantly lower VLDL (90 %), LDL (87 %), HDL (34 %) and total lipoprotein (54 %) than animals on the HF diet (Fig. 2). Our results showed that 1 % CA completely reversed the HF diet-induced increase of lipoprotein levels, and lowered VLDL, LDL, HDL and total lipoprotein levels back to the same levels as those of the chow diet group (Fig. 2).

### Cholesterol and lipid content of liver

**Fig. 3.**
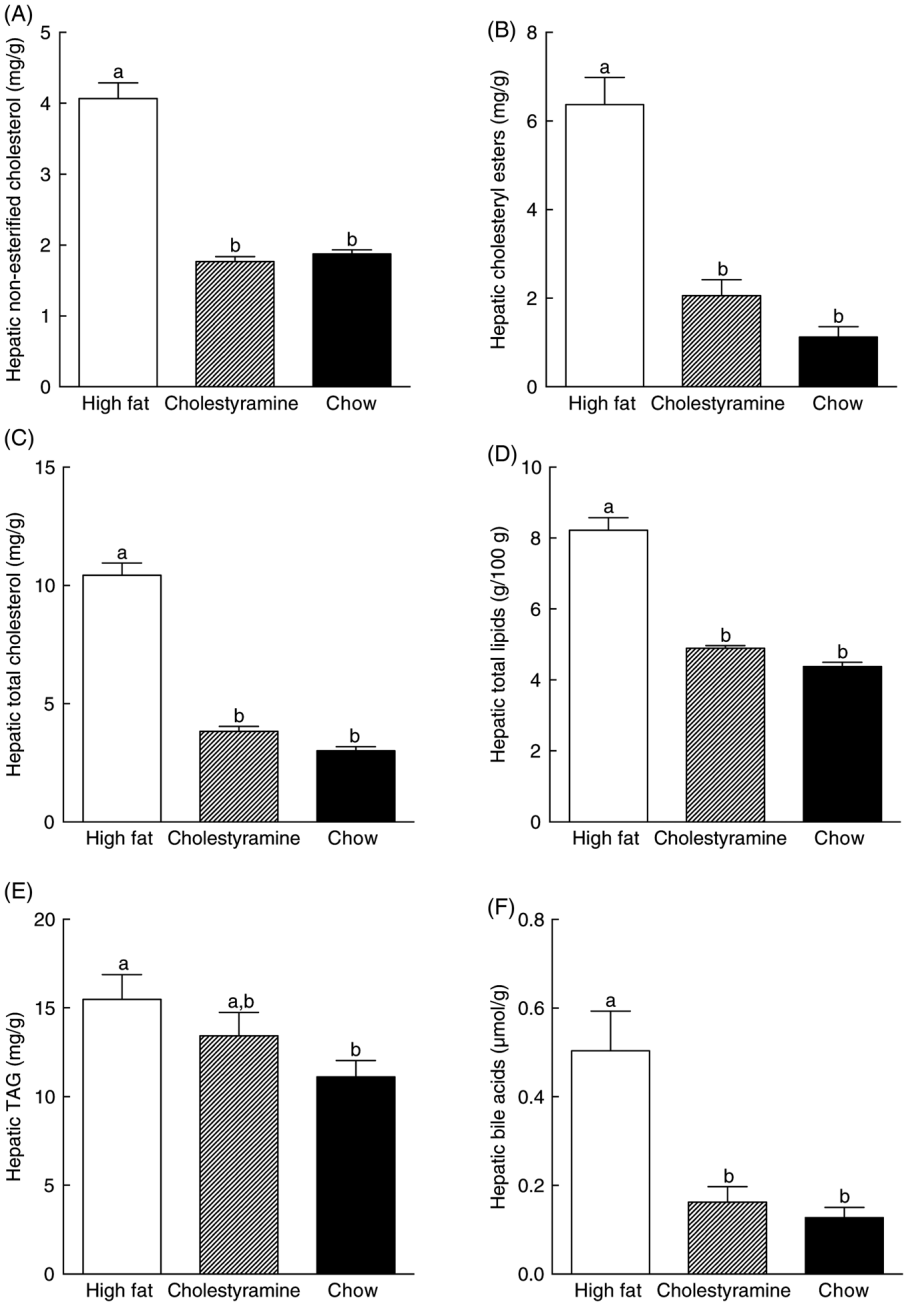
Effects of cholestyramine supplementation on hepatic cholesterol, TAG and bile acid levels. Livers were harvested from animals on different diets and hepatic lipid extracted and enzymically determined as described in Materials and methods. (A) Hepatic non-esterified cholesterol, (B) hepatic cholesteryl esters, (C) hepatic total cholesterol, (D) hepatic total lipids, (E) hepatic TAG and (F) hepatic bile acids. Values are means (*n* 10), with standard errors represented by vertical bars. ^a,b^ Mean values with unlike letters were significantly different (*P* ≤ 0·05).

In animals fed the HF diet, hepatic cholesteryl esters and non-esterified cholesterol increased 465 and 118 %, respectively, compared with those of animals on the chow diet, and cumulatively resulted in a 248 % increase in hepatic total cholesterol (Fig. 3). Hepatic cholesteryl esters and non-esterified cholesterol were lower in the HF + CA group (57 and 68 %, respectively) compared with the HF group (Fig. 3(A) and (B)). Overall, the total lipid content in the liver of HF diet animals was 88 % higher than those on the chow diet, and total lipid in the liver of the HF + CA group was 41 % lower than HF diet-fed animals, and at the same level as that of chow diet animals (Fig. 3(D)). Hepatic TAG level in the HF diet group increased 40 % compared with that of the chow diet group, while the HF + CA group showed a 14 % reduction in TAG level, although the differences did not reach statistical significance (Fig. 3(E)). Hepatic bile acid was 68 % lower in the HF + CA group than that of the HF group, which was similar to the bile acid level in the chow diet group (Fig. 3(F)).

### Cholesterol and bile acid content of faeces

**Fig. 4.**
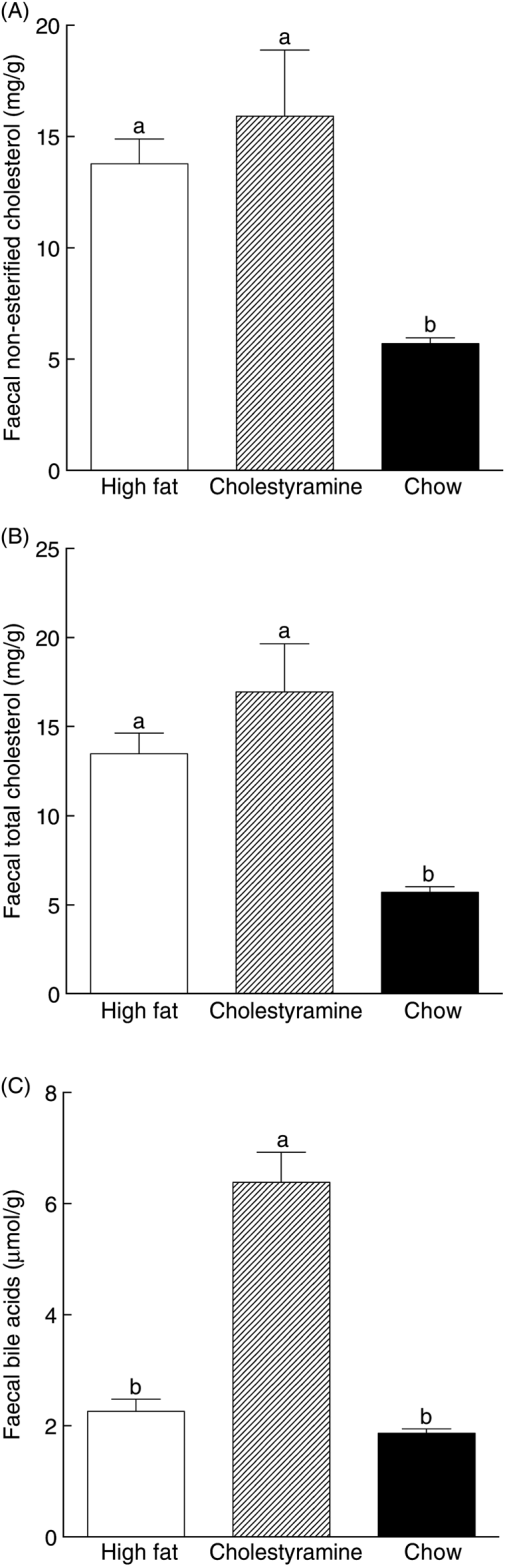
Effects of cholestyramine supplementation on cholesterol and faecal bile acid levels. Faecal samples were collected for 2 d for animals on different diets and faecal bile acid and total cholesterol were extracted and determined as described in Materials and methods. (A) Faecal non-esterified cholesterol, (B) faecal total cholesterol and (C) faecal bile acids. Values are means (*n* 10), with standard errors represented by vertical bars. ^a,b^ Mean values with unlike letters were significantly different (*P* ≤ 0·05).

A significant increase in faecal cholesterol was observed in HF groups compared with the chow diet group. There was no significant difference between the HF group and the HF + CA group in excretion of faecal non-esterified and total cholesterol (Fig. 4(A) and (B)). Compared with animals on the HF diet, the HF + CA group showed a 183 % increase in bile acid excretion, while there were no differences in faecal bile acid levels between the HF and chow groups (Fig. 4(C)).

### Relative expression of genes related to cholesterol and bile acid metabolism and ingenuity pathway analysis

**Fig. 5.**
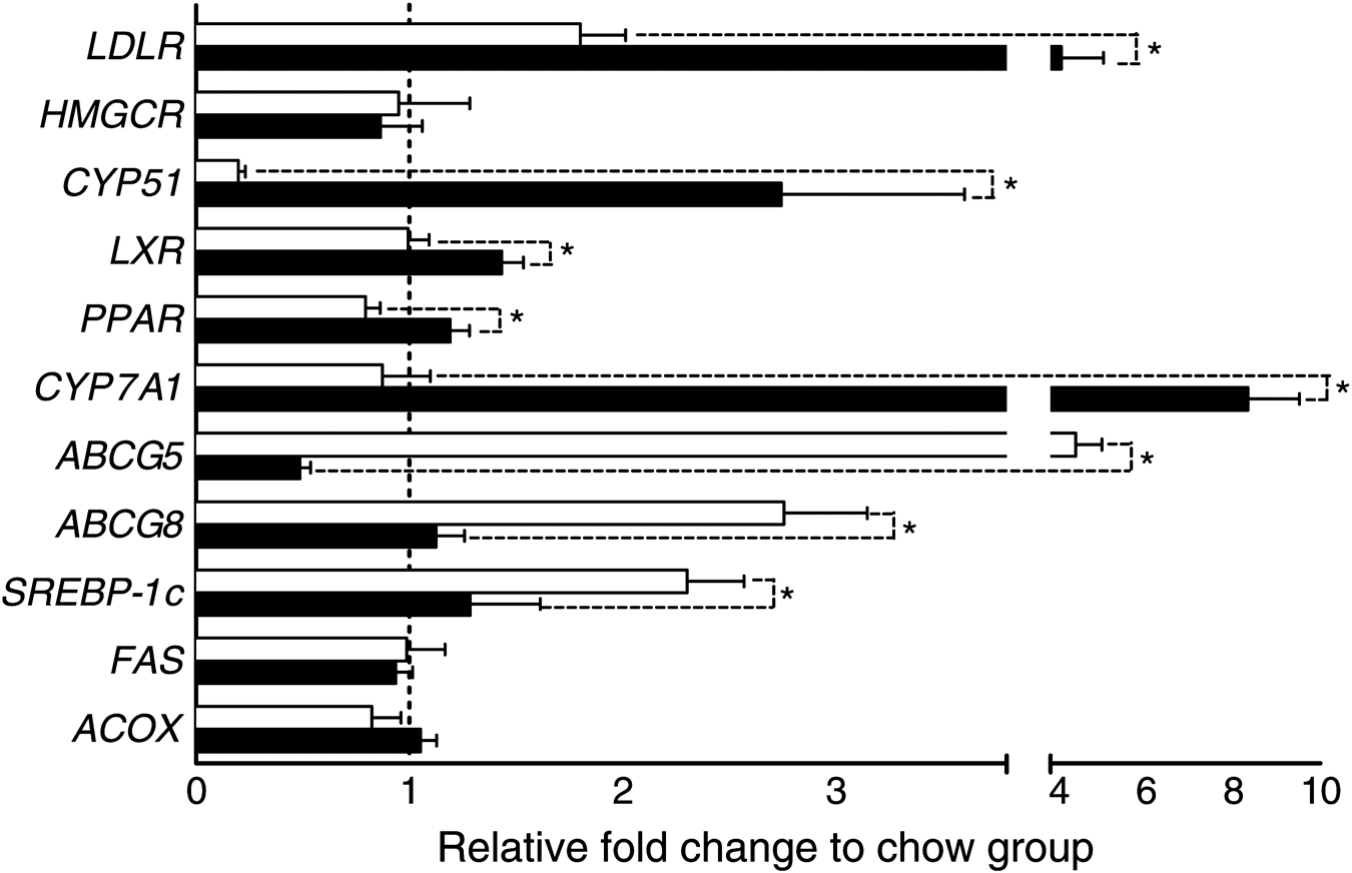
Effects of cholestyramine supplementation on hepatic mRNA expression levels. Livers were harvested from animals on different diets; total mRNA extracted and mRNA level were determined using real-time PCR as described in Materials and methods. Results are expressed as relative expression levels to the chow diet group (---). Values are means (*n* 10), with standard errors represented by horizontal bars. * Mean values were significantly different from each other (*P* ≤ 0·05). □, High-fat group; ■, cholestyramine-supplemented group. *LDLR*, LDL receptor; *HMGCR*, 3-hydroxy-3-methyl-glutaryl-CoA reductase; *CYP51*, lanosterol 14α-demethylase; *LXR*, liver X receptor; *CYP7A1*, cholesterol 7α-hydroxylase; *ABCG5/8*, ATP-binding cassette subfamily G member 5/8; *SREBP*, sterol regulatory element-binding protein; *FAS*, fatty acid synthase; *ACOX*, acyl-CoA oxidase.

To elucidate the metabolic pathways affected by changes in circulating cholesterol levels, expression of hepatic cholesterol, bile acid and fatty acid metabolism genes were determined. There were no differences in the LDL receptor mRNA expression levels between the HF group and the chow group, while the HF + CA group had a 126 % increase in LDL receptor expression (Fig. 5). Compared with the chow diet group, the hepatic mRNA level of the HMG-CoA reductase in the HF group, with or without CA, decreased about 45 % (Fig. 5). However, due to the variance between individual animals, the change did not reach statistical significance. Another rate-limiting enzyme in cholesterol synthesis, *CYP51*, was down-regulated 88 % in the HF group, while a 1280 % up-regulation was observed in the HF + CA group (Fig. 5).

Liver X receptor (LXR) α and PPARα are key factors involved in the regulation of cholesterol metabolism^(^^27^^,^^28^^)^. *LXRα* mRNA expression was up-regulated by 45 % in the HF + CA group, and the HF and chow diet groups showed similar expression levels (Fig. 5). *PPARα* expression was suppressed by 26 % by the HF diet, and the suppression was reversed in the HF + CA group (Fig. 5).

*CYP7A1* was increased by 855 % in the HF + CA group (Fig. 5), and the HF and chow diets showed similar expression levels of *CYP7A1*. The HF diet significantly elevated both *ABCG5* and *ABCG8* mRNA expression by 295 and 158 %, respectively (Fig. 5). Animals in HF + CA group expressed significantly lower levels of *ABCG5* (89 %) and *ABCG8* (60 %) mRNA as compared with that of the HF diet animals (Fig. 5), which were back to the same level as that of the chow diet group.

Animals on the HF diet had 98 % higher hepatic sterol regulatory element-binding protein (*SREBP*)-1c expression than animals on the chow diet (Fig. 5). Expression of *SREBP-1c* in the HF + CA group was 45 % lower and reduced to the same level as that of the chow diet group (Fig. 5). The mRNA levels of fatty acid synthase and acyl-CoA oxidase were not affected by the different diets (Fig. 5).

**Fig. 6.**
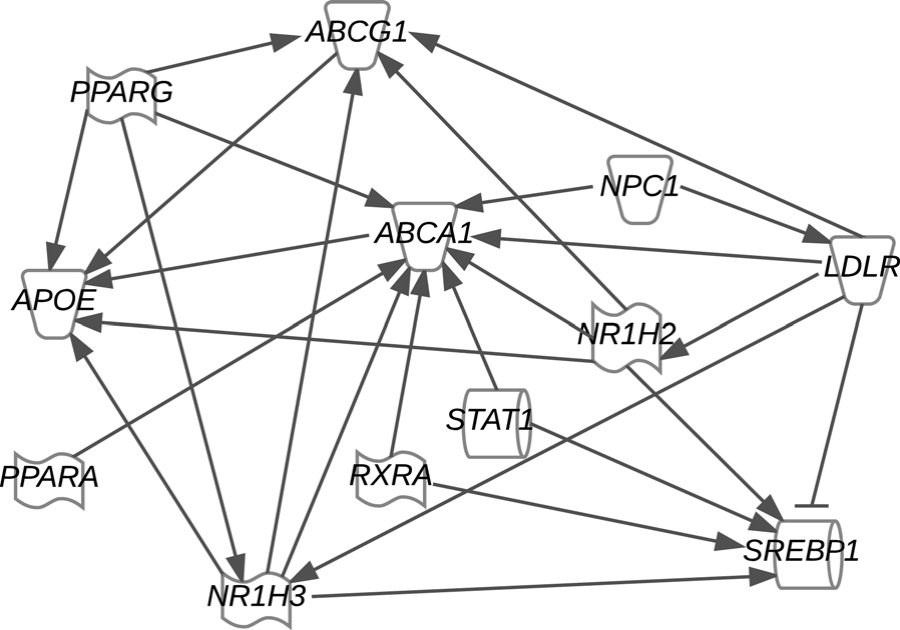
Ingenuity Pathway Analysis (IPA) of cholesterol and bile acid metabolism genes. Gene expression data were analysed using IPA core analysis and a network was generated. *ABCG1*, ATP-binding cassette subfamily G member 1; *PPARG*, PPAR-γ; *NPC1*, Niemann–Pick C1; *ABCA1*, ATP-binding cassette subfamily A member 1; *LDLR*, LDL receptor; *APOE*, apoE; *NR1H2*, gene for liver X receptor-β; *STAT1*, signal transducer and activator of transcription 1; *PPARA*, PPAR-α; *RXRA*, retinoid X receptor; *SREBP1*, sterol regulatory element-binding protein 1; *NR1H3*, for gene for liver X receptor-α.

IPA of the cholesterol and bile acid metabolism genes identified that reduction in circulating cholesterol activated multiple canonical pathways including LXR/retinoid X receptor (RXR) (*P* < 0·001), farnesoid X receptor/RXR (*P* < 0·001), thyroid hormone receptor/RXR (*P* < 0·001) and hepatic cholestasis (*P* < 0·001), and predicted RXRα (*P* = 0·001), LDL receptor (*P* = 0·005), Niemann–Pick C1 protein (NPC1) (*P* = 0·007), LXRβ (*P* = 0·010), and signal transducer and activator of transcription 1 (STAT1) (*P* = 0·011) as upstream regulators (Fig. 6). The pathway analysis also determined that inflammatory response (*P* < 0·038), CVD (*P* < 0·002) and metabolic disease (*P* = 0·010) were presented in the HF animals.

### Correlation between plasma lipoprotein, hepatic and faecal cholesterol and hepatic gene expression

Correlations of plasma lipoprotein concentration, hepatic lipid, cholesterol and bile acids, faecal cholesterol and bile acids and hepatic gene expression levels were examined to understand the relationship between molecular and physiological changes resulting from different circulating cholesterol levels (Tables 3 to 5). Plasma total lipoprotein (*r* −0·409) and HDL (*r* −0·525) were negatively correlated with LDL receptor expression, while bile acids (*r* 0·492) excretion correlated with LDL receptor expression level. In the cholesterol synthesis pathway, HMG-CoA reductase expression did not correlate with any physiological changes measured in this study; and *CYP51* was negatively correlated with liver weight (*r* −0·444), plasma lipoproteins (*r* −0·541) and hepatic cholesterol (*r* −0·427). *LXRα* and *PPARα*, the upstream regulators in cholesterol metabolism and synthesis, were also negatively correlated with plasma lipoproteins (*r* −0·559 and *r* −0·410, respectively), with *PPARα* negatively correlated with hepatic cholesterol (*r* −0·406) and positively with faecal bile acids (*r* 0·643), and *LXRα* negatively correlated with hepatic bile acids (*r* −0·432) and positively with faecal bile acids (*r* 0·472). In the cholesterol excretion pathway, *CYP7A1* expression was correlated with adipose tissue (*r* 0·446), plasma lipoproteins (*r* −0·523), hepatic non-esterified cholesterol (*r* −0·420) and faecal cholesterol (*r* −0·516), and most significantly faecal bile acids (*r* 0·824). *ABCG5* and *ABCG8* showed correlation with liver weight (*r* 0·470 and *r* 0·378), plasma lipoproteins (*r* 0·696 and *r* 0·508), hepatic cholesterol (*r* 0·763 and *r* 0·693) and TAG (*r* 0·409 and *r* 0·437). Additionally, *ABCG5* also correlated with hepatic and faecal bile acids (*r* 0·368 and *r* −0·477). In the fatty acid metabolism pathway, *SREBP-1c* was correlated with liver weight (*r* 0·392), plasma lipoproteins (*r* 0·497) and hepatic cholesterol (*r* 0·519). Fatty acid synthase did not show any significant correlation with any physiological changes in this study, and acyl-CoA oxidase was correlated with liver weight (*r* −0·374), VLDL (*r* −0·386), HDL(*r* −0·455) and total lipoprotein (*r* −0·433), and hepatic bile acids (*r* −0·392).

**Table 3.** Correlations between hepatic gene expression and liver, adipose weight and plasma lipoproteins

	Liver weight	Adipose weight	VLDL	LDL	HDL	Total lipoprotein
*LDLR*	−0·356	−0·077	−0·371	−0·247	−0·525**	−0·409*
*HMGCR*	−0·173	−0·123	−0·174	−0·120	−0·233	−0·188
*CYP51*	−0·444*	0·014	−0·468**	−0·371*	−0·658**	−0·541**
*LXRα*	−0·453*	0·106	−0·538**	−0·414*	−0·618**	−0·559**
*PPARα*	−0·336	0·170	−0·401*	−0·354	−0·396*	−0·410*
*CYP7A1*	−0·347	0·446*	−0·465**	−0·455*	−0·526**	−0·523**
*ABCG5*	0·470**	−0·312	0·580**	0·701**	0·620**	0·696**
*ABCG8*	0·378*	−0·269	0·420*	0·518**	0·446*	0·508**
*SREBP1*	0·392*	0·023	0·436*	0·384*	0·554**	0·497**
*FAS*	0·297	0·185	0·200	−0·087	0·197	0·086
*ACOX*	−0·374*	0·070	−0·386*	−0·359	−0·455*	−0·433*

*LDLR*, LDL receptor; *HMGCR*, 3-hydroxy-3-methyl-glutaryl-CoA reductase; *CYP51*, lanosterol 14α-demethylase; *LXR*, liver X receptor; *CYP7A1*, cholesterol 7α-hydroxylase; *ABCG5/8*, ATP-binding cassette subfamily G member 5/8; *SREBP*, sterol regulatory element-binding protein; *FAS*, fatty acid synthase; *ACOX*, acyl-CoA oxidase.

* Significant correlation (*P* < 0·05; two-tailed).

** Significant correlation (*P* < 0·01; two-tailed).

**Table 4.** Correlations between hepatic gene expression and hepatic lipid, cholesterol and bile acids

	Lipid content	Total cholesterol	Non-esterified cholesterol	Cholesteryl ester	TAG	Bile acids
*LDLR*	−0·194	−0·215	−0·321	−0·096	−0·146	−0·116
*HMGCR*	−0·206	−0·190	−0·138	−0·267	−0·096	−0·269
*CYP51*	−0·437*	−0·427*	−0·490**	−0·449*	−0·164	−0·332
*LXRα*	−0·123	−0·271	−0·245	−0·353	0·216	−0·432*
*PPARα*	−0·456*	−0·406*	−0·545**	−0·332	−0·013	−0·240
*CYP7A1*	−0·312	−0·346	−0·420*	−0·223	−0·182	−0·154
*ABCG5*	0·893**	0·763**	0·836**	0·628**	0·409*	0·368*
*ABCG8*	0·826**	0·693**	0·842**	0·478**	0·437*	0·167
*SREBP1*	0·551**	0·519**	0·615**	0·361*	0·312	0·079
*FAS*	−0·135	−0·042	0·048	−0·188	0·105	−0·248
*ACOX*	−0·243	−0·251	−0·200	−0·321	−0·049	−0·392*

*LDLR*, LDL receptor; *HMGCR*, 3-hydroxy-3-methyl-glutaryl-CoA reductase; *CYP51*, lanosterol 14α-demethylase; *LXR*, liver X receptor; *CYP7A1*, cholesterol 7α-hydroxylase; *ABCG5/8*, ATP-binding cassette subfamily G member 5/8; *SREBP*, sterol regulatory element-binding protein; *FAS*, fatty acid synthase; *ACOX*, acyl-CoA oxidase.

* Significant correlation (*P* < 0·05; two-tailed).

** Significant correlation (*P* < 0·01; two-tailed).

**Table 5.** Correlation between hepatic gene expression and faecal cholesterol and bile acids

	Total cholesterol	Non-esterified cholesterol	Bile acids
* LDLR*	0·173	0·120	0·492*
* HMGCR*	−0·390	−0·391	−0·263
* CYP51*	0·082	0·017	0·395
* LXRα*	0·103	0·084	0·472*
* PPARα*	0·121	0·062	0·643**
* CYP7A1*	0·516*	0·484*	0·824**
* ABCG5*	−0·084	−0·005	−0·477*
* ABCG8*	0·019	0·095	−0·316
* SREBP1*	−0·048	0·044	−0·363
* FAS*	−0·009	0·008	−0·076
* ACOX*	−0·099	−0·092	0·041

*LDLR*, LDL receptor; *HMGCR*, 3-hydroxy-3-methyl-glutaryl-CoA reductase; *CYP51*, lanosterol 14α-demethylase; *LXR*, liver X receptor; *CYP7A1*, cholesterol 7α-hydroxylase; *ABCG5/8*, ATP-binding cassette subfamily G member 5/8; *SREBP*, sterol regulatory element-binding protein; *FAS*, fatty acid synthase; *ACOX*, acyl-CoA oxidase.

* Significant correlation (*P*<0·05; two-tailed).

** Significant correlation (*P*<0·01; two-tailed).

### Regression analysis of hepatic lipid concentration, total lipoprotein concentration, faecal bile acids concentration and selected hepatic gene expression

**Fig. 7.**
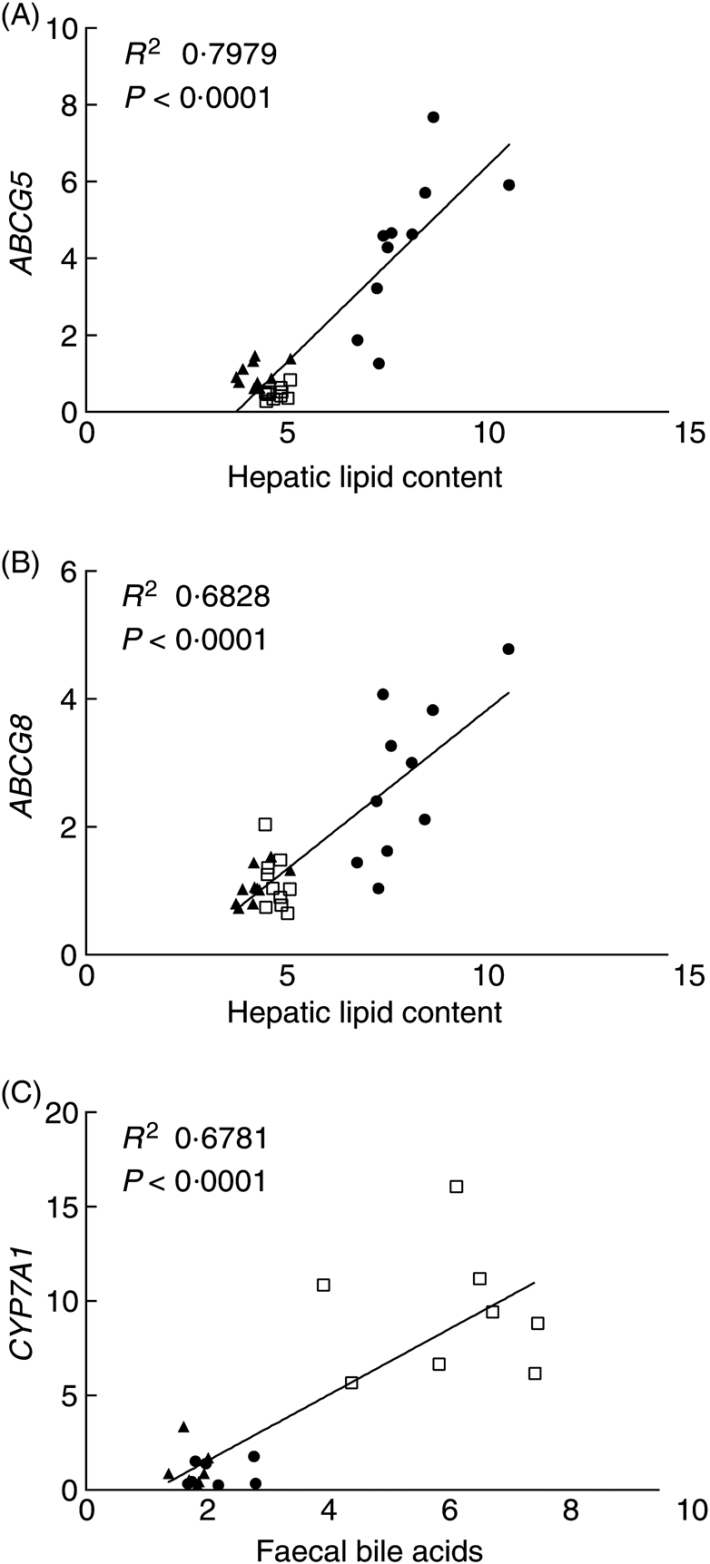
Regression analysis of hepatic gene expressions and physiological changes. Linear regression analysis of (A) ATP-binding cassette subfamily G member 5 (*ABCG5*), (B) ATP-binding cassette subfamily G member 8 (*ABCG8*) and hepatic lipid content, and (C) cholesterol 7α-hydroxylase (*CYP7A1*) and faecal bile acids. ●, High-fat group, ☐; high-fat + cholestyramine group; ▲, chow group.

Regression analysis was performed to determine the relationship between concentration changes of hepatic lipids, total lipoprotein and faecal bile acid, and hepatic gene expression levels. We focused on representative hepatic genes *ABCG5/8*, *CYP7A1* and *CYP51*. There was a linear increase of *ABCG5* and *ABCG8* mRNA levels with hepatic lipid concentration (*R*^2^ 0·7979 and 0·6828, respectively; Fig. 7(A) and (B)). Increased faecal bile acid was associated with increased *CYP7A1* mRNA levels (*R*^2^ 0·6781; Fig. 7(C)). *CYP51* mRNA levels, linear regression to total lipoprotein concentration showed an *R*^2^ of 0·2926 (*P* = 0·002), to hepatic total cholesterol showed an *R*^2^ of 0·1823 (*P* = 0·0186), and an *R*^2^ of 0·1559 (*P* = 0·069) to hepatic bile acids.

## Discussion

This study identified *CYP51* as a gene responsive to changes in circulating cholesterol induced by the HF diet and CA in hamsters. CYP51 converts lanosterol to cholesterol, and is a critical enzyme in the *de novo* synthesis of cholesterol. Increased circulating cholesterol induced by the HF diet significantly lowered *CYP51* mRNA expression in the liver (Fig. 5), which was consistent with a previous study using a Sprague–Dawley rat model^(^^29^^)^. In contrast, decreased circulating cholesterol induced by CA significantly up-regulated *CYP51* expression in the liver (Fig. 5). Previous studies mostly focused on HMG-CoA reductase's role in cholesterol regulation, but discrepancy existed in the literature, which may result from the different models (human, mouse and rat) used in these studies^(^^10^^,^^30^^,^^31^^)^. In the present study, HMG-CoA reductase, the classic rate-limiting enzyme involved in cholesterol synthesis pathway upstream of CYP51, was not affected by the HF or HF + CA diet, and independent of circulating cholesterol levels (Fig. 5). We also observed that *CYP51* expression levels were correlated with liver weight, plasma VLDL, LDL, HDL, and hepatic non-esterified cholesterol and cholesteryl ester (Tables 3 and 4). Previous research characterised that CYP51 can respond to circulating cholesterol through SREBP, which are regulated by a lipid-sensor mechanism^(^^32^^)^. Three clustered regulatory elements (GC box, cAMP-response elements, and sterol regulatory element) were found in the proximal promoter region of CYP51. CYP51 was shown to also respond to the AMP-dependent signalling pathway in the absence of mature SREBP and a single cAMP-response element is sufficient to mediate an immediate early transactivation of the *CYP51* gene^(^^33^^)^. Based on our results, *CYP51* appeared to be more sensitive to fluctuation of circulating cholesterol. Induction of *CYP51* in HF + CA animals reflects that liver cells can sense decreases in cholesterol levels and up-regulate *CYP51* to compensate for this change. Induction of *CYP51* might thus significantly increase *de novo* cholesterol synthesis providing sufficient substrate, namely lanosterol, without the involvement of HMG-CoA reductase. Hence, CYP51 may deserve more attention as a potential target in terms of hypercholesterolaemia treatments, and administration of CYP51 inhibitor, in combination with HMG-CoA reductase inhibitor, may achieve a further reduction in disease-state circulating and hepatic cholesterol levels.

Changes in hepatic lipid content and faecal bile acid concentration and changes in *ABCG5/8* and *CYP7A1* mRNA levels, respectively, could be fitted to a linear model. This suggested a direct relationship between the gene expression and hepatic lipid and faecal bile acid levels. Increases in the excretion of bile acids in HF + CA animals led to de-repression of *CYP7A1* expression in the liver, which is consistent with previous studies^(^^34^^)^. In contrast, changes in *CYP51* mRNA levels could not be fitted to a linear model with plasma lipoprotein levels, hepatic total cholesterol or bile acids. A higher-order polynomial model was required to get a better fit. Though *CYP51* showed significant correlation with plasma lipoprotein concentration, hepatic lipid content and cholesterol level, none of them was a dominant factor in the regression analysis. These results indicated that the regulation of *CYP51* expression may be due to multiple factors. *CYP51* was identified as an immediate early response gene in the SREBP regulation pathway^(^^33^^)^. Previous reports also showed up-regulation of *CYP51* by phytochemical or viscous carbohydrate polymer treatments^(^^3^^,^^4^^)^. Others also reported that the regulation of *CYP51* expression may be subject to multiple pathways and upstream regulators^(^^32^^,^^33^^,^^35^^)^. Our results were consistent with this notion but further study is needed to elucidate the precise mechanisms.

CA is known to bind bile acids and increase their excretion, but faecal cholesterol level has not usually been reported. A previous study reported that CA treatment induced an increase in faecal cholesterol excretion in a mouse model^(^^36^^)^. In our study, using the hamster as a model, the HF diet was shown to induce an increase of faecal cholesterol excretion, while faecal cholesterol (non-esterified form or esters) was not affected by CA treatment (Fig. 4(A) and (B)). This result confirms a previous study of dietary CA in hamsters^(^^37^^)^. Additional studies are needed to confirm these observations in human subjects.

In the present study, supplementation of 1 % CA in a HF diet for 5 weeks was shown to significantly reduce circulating lipoprotein, hepatic cholesterol and lipid levels to a level similar to animals on a chow diet. The recommended daily dose of CA is 4–8 g or about 0·4 to 0·8 % in the approximate 958 g average food intake by the US population^(^^38^^)^. Thus, 1 % CA in hamster diets is comparable with that suggested for humans. The 1 % CA supplementation was able to reduce about 87 % VLDL + LDL in hamsters on a HF diet for 5 weeks (Fig. 2). Suckling *et al.*^(^^39^^)^ conducted a 1-week study and determined that 2 % CA achieved a 55 % decrease in VLDL + LDL in hamsters. Our results suggest that length of treatment might be as important a variable as dose.

CA in the HF diet reduced hepatic non-esterified cholesterol and cholesteryl esters, as well as bile acid, to a similar level to the chow diet group (Fig. 3). Previous studies have shown that CA, psyllium and hydroxyl-propylmethylcellulose feeding increased bile acid excretion and reduced non-esterified and esterified hepatic cholesterol^(^^3^^,^^37^^)^. Animals consuming CA excreted significantly more bile acids, while those on just HF and the chow diet showed similar amount of bile acids excretion as well as their *CYP7A1* level, regardless of cholesterol intake (Fig. 4(C)).

Increase in mRNA expression of *CYP7A1* (Fig. 5) was strongly correlated with faecal bile acid excretion (Table 5), and also to the weight of adipose tissue and plasma lipoproteins (Table 3). *LXRα*, which serves as a dominant *CYP7A1* activator in the liver, exhibited a significant increase upon CA consumption (Fig. 5) and was also significantly correlated with faecal bile acids excretion (Table 5). Our finding confirmed the increase of *LXRα* previously reported in mouse and rat models^(^^36^^,^^40^^)^. These results supported that CA treatment regulates *CYP7A1* through an *LXRα*-dependent pathway. Coupled with the bile acid/CYP7A1 correlation discussed above, CA regulation of *CYP7A1* may be through both pathways. Lower bile acid concentration de-repressed *CYP7A1* transcription while an increase in *LXRα* also allowed for increased transcription.

The increased expression level of *ABCG5/8* was a major factor towards maintaining hepatic lipid and cholesterol levels (Fig. 5, Table 4). *SREBP-1c*, a transcription factor located upstream of fatty acid biosynthesis genes, expression was lowered by reduced circulating cholesterol (Fig. 5) and indicates that cholesterol level may mediate fatty acid metabolism. As shown in the IPA predicted upstream network in Fig. 6, *SREBP-1c* can be suppressed by activation of LDL receptor, which matched our observation in the HF diet group in this study (Fig. 5). On the other hand, pathway analysis indicated that *SREBP-1c* is regulated by multiple upstream transcriptional factors, including *LXR*, *RXRα* and *STAT1* (Fig. 6). Similar changes in *ABCG5/8* and *SREBP-1c* had been observed in rats and mice treated with CA^(^^31^^,^^41^^)^, and in the intestine of hamsters fed chow supplemented with 4 % (w/w) CA and 0·15 % (w/w) lovastatin for 2 weeks^(^^42^^)^. A previous study in human subjects treated with CA (8 g twice per d) reported no change in *ABCG5/8* and *SREBP-1c*. Differences between this human study and animal studies might be due to the limited term (3 weeks) and small sample number of subjects (*n* 5)^(^^43^^)^ and warrant further study.

In summary, our study using the hamster model provided additional molecular insights into the influence of circulating cholesterol on hepatic cholesterol metabolism pathways during hypercholesterolaemia produced by diets high in fat and cholesterol. *CYP51* was identified as a marker of cholesterol homeostasis that may be regulated by multiple pathways. Reduced circulating cholesterol levels did not affect lipid metabolism genes (fatty acid synthase and acyl-CoA oxidase) or adipose weight. Additionally, *CYP7A1* was identified to be regulated through an *LXR*-mediated pathway.
